# How Does Social Currency Influence Prosocial Behavior? The Role of Collective Self-Esteem and Communication Network Heterogeneity

**DOI:** 10.3389/fpsyg.2021.672505

**Published:** 2021-09-10

**Authors:** Xiaoxiao Gong, Zuoliang Ye, Binjie Liu, Shengxian Yu, Yufei Yan

**Affiliations:** ^1^School of Business Administration, Southwestern University of Finance and Economics, Chengdu, China; ^2^School of International Business, Southwestern University of Finance and Economics, Chengdu, China; ^3^School of Business Administration, South China University of Technology, Guangzhou, China

**Keywords:** social currency, prosocial behavior, collective self-esteem, communication network heterogeneity, social networks

## Abstract

The social currency, the existing potential resource in the social networks and communities of an individual, has become more significant in the era of information technology. Meanwhile, the rapid development of Internet service, especially its application on mobile devices, brings many new contents of prosocial behaviors (PBs), which benefits both individuals and communities. Specifically, social currency plays a positive role in promoting PB, forming positive personalities, promoting positive social adaptation, and contributing to human survival and social development. However, the theoretical research in this field still lags far behind the development of practice, and the research on the impact of social currency on PB remains exceedingly scarce. Grounded in the social exchange theory (SET), the present study collects a total of 497 WeChat user questionnaires, constructs a model for the influence mechanism of social currency on PB, and tests the hypothesis through hierarchical regression. According to the results obtained, it was found that the social currency is positively associated with PB, and users with a more social currency are more likely to act prosocially on their own. The PB of an individual is then influenced by the social currency generated through collective self-esteem (CS) so that the perception of CS can be considered as a mediating variable. Moreover, the communication network heterogeneity (CNH) moderates the above relationship, and the degree of heterogeneity will have different effects on the relationship of CS. The obtained conclusions enrich the previous theoretical results of PB and provided new insights for social managers to enhance the prosocial-related behaviors for the group, organization, and society.

## Introduction

Powered by advanced technology and the borderless procrastination magnet of social networks, social media usage has increasingly proliferated (Chen et al., 2020; Gong et al., 2020b). The users of social media can express their views and communicate more efficiently (Chen et al., 2019), which has reshaped their social network as their ideas and social behavior are influenced by the social network. As one common form of social behavior, prosocial behavior (PB) is defined as a voluntary behavior in which an individual is willing to devote time, energy, and resources to benefit others, including the group and the society (Eisenberg et al., 2015; Memmott-Elison et al., 2020; Thielmann et al., 2020). For instance, a user of WeChat (i.e., one of the most popular social media platforms of the world, like WhatsApp and Facebook) has his profile photo plastered with a mask to remind himself/herself and advise others to protect well-during COVID-19. Another example, “a cup of milk tea in autumn” has recently become popular among WeChat Moments (i.e., a social platform provided rich media functions; Zhang et al., 2017). In this case, people click the “Likes” response to endorse the content shared by friends or receive “Likes” from friends (Kosinski et al., 2015; Wang et al., 2018), which remain appealing to every individual to pour attention to the emotional exchange with their friends and families. Similarly, existing studies have explored the research on the implicit association between Facebook users and PBs, and the Facebook users may be changing their relationship with social networks (Ferenczi et al., 2017; Villacampa et al., 2018).

Considering the importance of PB in building a harmonious network environment and exerting a positive role in the social network, it is of great necessity to investigate the related influencing factors and mechanisms to cultivate individual PB and promote its positive development. More precisely, the willingness Social currency has proven to be an effective way for individuals to strengthen ties with others in their social networks, enhance reciprocity, and strengthen social cohesion (Tandoc et al., 2020). It means the totality of actual and potential social interactions that occur primarily on social media and other online platforms, as well as social networks and groups that exist in both the virtual and physical worlds (Lobschat et al., 2013; Trudeau and Shobeiri, 2016) of an individual to share information with others contributes to improving products or ideas. Just like dollars can be used to purchase goods and services, social currency can garner more favorable comments and impressions from family, friends, and colleagues. This explains why we frequently see people sharing as much content as possible to enhance their appearance to create a pleasurable image in WeChat Moments. In this sense, the social interaction among the users leads to information acquisition, knowledge exchange, and other social benefits, such as self-identity recognition and realization of PB. As a result, it is worthwhile to examine how social currency influences PBs from the perspective of social exchange.

By and large, previous research has examined the factors and mechanisms that influence PB, including internal motivation (Christner et al., 2020; Gherghel et al., 2020; Shukla and Kark, 2020), community forces (Vives and FeldmanHall, 2018), and social support (DuBois et al., 2002). However, previous research has overlooked the critical role of social currency in converting individual motivation to social behavior. Few scholars have examined to find how the PB of social currency users is influenced in social networks. Meanwhile, previous studies explored the mediating mechanism of self-esteem in PBs (Guo et al., 2018b; Gao et al., 2019) while they did not refine self-esteem into collective self-esteem (CS), an essential communication tool to enhance the senses of membership and group involvement of individuals, which should be a vital factor in PB. Besides, although the social currency of an individual is prone to stimulate favorable and beneficial behaviors, the extent to which it functions is affected by specific exchange characteristics. Communication network heterogeneity (CNH), the degree to which an individual communicates with others and demonstrates the difference (Kim and Kim, 2019) will strengthen the process from individual attitude to social psychology (Woolley et al., 2010). It indicates that the more community communication appears, the richer the network becomes, and the more significant is the performance of the impact. However, there is still no relevant research to test the moderating effect of CNH on the relationships among the social currency and its related factors.

To bridge these gaps, this study examines the impact of the social currency of users on their PBs in the specific context of WeChat. By developing a theoretical model that incorporates both the mediating role of CS and the moderating role of CNH, the “black box” and boundary conditions of the social currency of the individual that influence the PB mechanism are revealed. Therefore, the expected results of our study might contribute much to the existing knowledge on individual PB in a social network.

The possible contributions of this study include the following threefold outcomes. To begin, we assessed the extent to which social currency encourages individuals to act with PB. Social currency may increase the willingness of an individual to contribute to society if the social currency is circulated more widely. This means that those with greater feedback and comments in social networks may enable individuals to have great experiences and even inspire them to alter their lifestyle, to follow the social models in a better manner. Second, we examined the underlying processes of social currency in social networks impacting PB, that is, the mediating effect of CS. When we interact with others, it increases our sense of self-worth, resulting in more generous conduct. The findings acquired by social managers can be used to boost the CS of the users. In the third part, we explained how boundary conditions affect the relationship. When the communication network is more heterogeneous, the social currency of the individuals affects CS, and their social currency can help them achieve individual prosocial goals. In a diverse environment, individuals can communicate with one another, listen, and take part in the activities in the social networks of others. In addition, community members will benefit from more meaningful connections and vigorous encounters that would help build positive social perceptions and lay the foundation for individuals to feel more confident and empowered.

This study includes five parts. The first section generally illustrates the current research. The second part introduces the theoretical background and hypothesis development. The following parts mainly deal with the methodology, which also presents an analysis of the empirical results. The last part includes the conclusion, limitations and directions of future studies.

## Literature Review

### Social Currency

In social networks, the social currency has remained a topic of concern for years. Bourdieu (1977) coined the term “social currency” to identify all the tangible and intangible resources. Individuals express what they believe makes them appear attractive. In other words, they communicate just what they feel which would help them preserve or improve their reputation among their peers. Social currency, as previously defined in the literature, may be divided into six different aspects that impact the behavior of the users on social media sites, that is, conversation, advocacy, information, affiliation, utility, and identity (Lobschat et al., 2013; Mishra and Singh, 2021). The conversation is the practice of exchanging thoughts and opinions in an informal setting between two or more persons (Mishra and Singh, 2021). Advocacy is an active effort by users to recommend and promote meaningful and valuable things that they agree to with others in their social networks. Information is the informational value of the content shared between the users and other social media users. Affiliation refers to the emotional attachment and sense of belonging that the users share with others in their social networks. Utility refers to the impact of the value of information on personal growth as mentioned previously by Lobschat et al. (2013). Identity refers to the way individuals present and express their personality in social networks. The definition of concept was modified to reflect the current work object. When individuals decide to disclose knowledge, they jeopardize their reputation and “spend” social currency (Mosley, 2021). Berger (2016) expanded on this premise by asserting that the social currency capitalizes on the social desire of the people to accomplish the goal of word-of-mouth communication. Additionally, social currency is viewed as a critical component of brand marketing (Lobschat et al., 2013; Trudeau and Shobeiri, 2016; Kesgin and Murthy, 2019). Consumers can gain social benefits from other users through communication *via* interactive comments and replies.

### Prosocial Behavior

Prosocial behavior refers to all behaviors that benefit others and society, such as helping, sharing, humility, cooperation, self-sacrifice, or individual voluntary behaviors that benefit others and promote interpersonal coordination (Penner et al., 2005). PB necessitates inter-individual relationships and communication in social contexts (Guo et al., 2018a). Recently, PB has attracted the attention of many researchers (Schroeder and Graziano, 2015; Malonda et al., 2019; Goh et al., 2021; Jin et al., 2021), which mainly focus on the role of PBs in promoting social cohesion and mitigating destructive behaviors (Malti and Dys, 2018; Baldassarri and Abascal, 2020). Besides, quite a few scholars have also explored the influence mechanism of implicit and explicit attitudes in PB (Weber et al., 2020), as well as its connection with voluntary behavior (Clary et al., 1998), life satisfaction, and self-esteem (Zuffianò et al., 2018). PBs, as a phenomenon related to emotions, situations, and cognitive factors (Martí-Vilar et al., 2019), not only enhance individual attitudes and performance but also promote innovation and improve effectiveness (Henttonen et al., 2016), which reveals a mutual-reinforcing multi-lever relationship. To put it in another way, a particular social and natural environment has an impact on PBs. Individuals are more likely to engage in PBs when they perceive an atmosphere of friendliness, harmony, and mutual assistance. As a result, elucidating the process behind PB becomes both intriguing and significant.

### Collective Self-Esteem

Collective self-esteem is defined to illustrate the degree to which an individual positively evaluates a social group (Kim and Kim, 2019). It places more emphasis on the sense of self-worth and self-esteem of a person. Suppose an individual develops CS, or positively feels or accepts shared group identity, strengths, and abilities. In that case, the individual can obtain a positive sense of self-worth from group members (Crocker and Luhtanen, 1990). The research conducted by Kim and Kim (2019) found that students who often use social media to talk about sports topics with various people in campus life have higher college community identity and CS. In addition, evidence has appeared to prove that the use of social media is generally associated with the CS of an individual (Gangadharbatla, 2008). For instance, Zhang et al. (2011) demonstrated that CS is a vital antecedent variable for social media usage, which can be regarded as an essential communication tool to enhance the senses of membership and group involvement of individuals. To this end, this study introduces CS as a mediating variable to uncover the “black box” of the influence of social currency on PB.

### Communication Network Heterogeneity

When the interaction of heterogeneous individuals on social media platforms produces meaningful social consequences, the concept of such interaction is termed, CNH (Kim et al., 2020). Existing research has reported that CNH influences the social behavior of social media users (Kim and Kim, 2019). In particular, the development of social media enables users to communicate or interact with others conveniently (Ellison et al., 2007). Due to the meaningful social consequences of interaction with heterogeneous opinions (Kim et al., 2013), a slice of studies has examined whether and how the usage of social media works on the interaction and communication among heterogeneous populations, which is known as CNH (Kim and Chen, 2015). For individuals with high CNH, they can share their ideas, learn about new ideas that they are unfamiliar with, and get inadvertently exposed to different information on the network or social media platforms to exchange different views with others, thus possessing more opportunities to recognize the heterogeneous perspectives of others (Brundidge, 2010; Kim et al., 2013). On the contrary, individuals with a low degree of heterogeneity in communication networks will fragment and polarize society, thus constituting a serious threat to democracy (Tewksbury and Rittenberg, 2012; Choi and Lee, 2015). The current study is designed to explore the moderating role of the heterogeneity in communication networks among social currency, CS, and PB.

## Theoretical Background and Hypotheses

### Social Currency and PB

Social currency is the concept of social economy, which was used to measure the propensity of the user to share brand-related content at first (Lobschat et al., 2013; Kesgin and Murthy, 2019), and then extended to measure the self-orientation of social media users (Wu and Ma, 2017; Tandoc et al., 2020). Social exchange theory (SET) provides a useful lens to comprehend the social interaction among people and the relationship established through interaction from the perspective of interest exchange. According to SET, individuals and groups establish the interactive relationship of exchange resources in the exchange situation with social characteristics (Ap, 1992), and actors obtain valuable returns or benefits in the social exchange. Additionally, it can also be said that social currency indicates that users generate or forward in-depth thought content, novel ideas, little-known secrets, as well as other attractive content to obtain a sense of identity and connection. A quintessential example should be cited that news information released on social media is a kind of social currency, exerting an important role in promoting the users to shape positive moral habits and civic behaviors (Tandoc et al., 2020). Not only the individual, but the groups can also increase their value, shape their image, and experience through the effective use of social currency (Trudeau and Shobeiri, 2016). For example, studies have shown that social currency is a driver of potential behavior change (Trudeau and Shobeiri, 2016) and exerts a positive role on brand experience, brand loyalty, and brand trust (Lobschat et al., 2013; Kesgin and Murthy, 2019). Moreover, we should at least nod to other important factors beyond social currency that substantially impact choices to be engaged in PB—for example, personal ideology (Schlenker, 2008) and personality (Hilbig et al., 2014). According to SET, it may be further extended that people could comment and share content through WeChat Moments to generate interactive value, such as obtaining social support, getting self-identity recognition, and exhibiting more PBs. These functional, relational, emotional, and substantive values may continuously enhance the quality of the relationship between the users and other subjects, which can ultimately produce favorable attitudes and behaviors for society.

Thus, based on the above discussion, this study assumes:

**Hypothesis H1**. *Social currency in social networks has a positive impact on PB*.

### The Mediating Role of CS

Collective self-esteem is the degree to which individuals perceive that they generally positively evaluate their social group (Cooper et al., 2021), better reflecting the psychological state of the users as community members. When the out-group threatens the realistic interests or cultural security of the in-group, it will influence its CS. People believe that online social interaction is a way to share knowledge and cultivate new relationships based on shared interests (Mesch and Talmud, 2006). The information transmitted through social currency can lower the perceived risks of the users caused by information asymmetry, increase collective trust, and form CS. Crocker and Luhtanen (1990) argued that CS was an evaluation of the group by members. Therefore, CS could also be considered as an in-group attitude. Previous studies have revealed that the CS of the individuals plays a mediating role between social support and psychological and behavioral adaptation (DuBois et al., 2002). Gangadharbatla (2008) demonstrated that CS positively affected the attitude toward social responsibility, which also exerted a partial mediating effect. People tend to engage in online interactions because they need a sense of belonging, and the use of social media enhances their sense of membership and team engagement. By performing the above analysis, users communicate with the platform and others to form social currency, creating and sharing information through social platforms, forming group self-esteem for the online communities and organizations to which they belong, standardizing their words and behaviors, and generating a series of PBs. The following hypothesis is formulated correspondingly:

**Hypothesis H2**. *Collective self-esteem plays a mediating role between social currency and PB in social networks*.

### The Moderating Role of CNH

The judgment of the people and recognition of their behavior abilities generate a dominating role in the self-regulation system. As the self-regulating mechanism changes the individual behavior, CNH plays a vital role. The possible reasons include that CNH may promote the interaction with others, and establish and maintain social relations (Woolley et al., 2010; Kim et al., 2013), making their social currency appreciation, which can simultaneously improve the perceived social skills and confidence, thus further being integrated into the surrounding social environment (Yang et al., 2016).

Earlier research suggested that the makeup of social networks can impact opinion strength (in both homogeneous and heterogeneous networks; Tormala and Petty, 2002; Levitan and Visser, 2008; Wojcieszak, 2010). When users have high CNH, they are more willing to communicate and share with others in the community within a high frequency (Jordan et al., 2011), which will deepen their understanding of the team, reduce uncertainty or doubts about others, form a high sense of identity and belonging to the collective, and then develop into CS as well as actively maintain the honor and reputation of the CS (Knobloch et al., 2010; Kim and Kim, 2019). Consequently, at a high level of CNH, social currency perception is likely to be taken as an important driving force for forming CS. In other words, CNH is conducive to transforming social currency perception in social networks into CS. If users do not have substantial CNH, they will doubt the truth of the social currency, more likely encountering difficulties in changing their personal behavior, and possibly leading to a decrease in their CS. Therefore, the following hypothesis is promoted.

**Hypothesis H3**. *Communication network heterogeneity plays a positive moderating role between social currency and CS in social networks*.

### The Moderated Mediating Role of CNH

Drawing upon SET leads to a process of communication and interaction among users (Ap, 1992). When users interact on social networks, even if users with a high level of CNH have a lower willingness to use social currency, they can still realize the importance of CS to their social currency. As a result, out of recognition and reward for collective identity, such users will make great attempts to face up to their collective identity and put the resources in their social currency into the social network, aiming to have more resources to realize the transformation from individual to collective thinking, thus achieving a higher degree of CS and realizing more PBs. Based on the above analysis, the CNH moderates the impact of social currency on CS, and CS mediates the relationship between social currency and PB. Therefore, it can be further inferred that the mediating effect of CNH on CS between social currency and individual PB may also generate a moderating effect. In the present study, compared to the low-level CNH, the high-level CNH on CS is more vulnerable to the impact of social currency. Consequently, the following assumptions are put forward:

**Hypothesis H4**. *The higher the CNH is, the stronger is the mediating role of CS between social currency and individual PB, and vice versa*.

**Figure 1:**
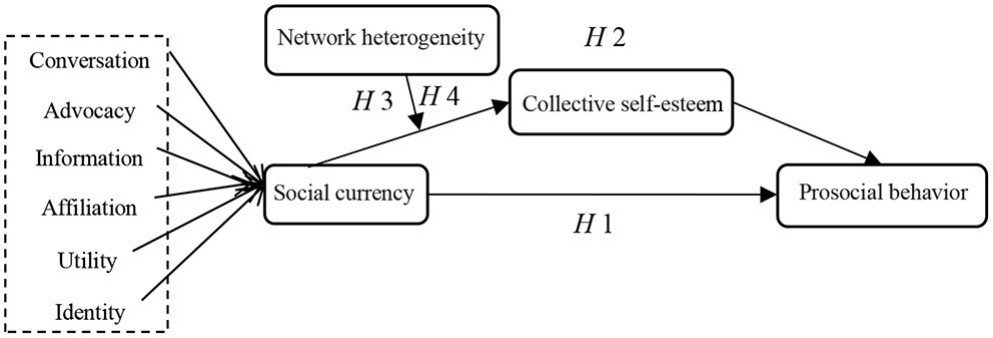
Conceptual model of social currency (SC) and prosocial behavior (PB).

By combining Hypotheses 1, 2, 3, and 4, we propose a moderated mediation model, as illustrated in Figure 1, to test the relationship between social currency and PB. The model considers CNH as a mediator and CS as a moderator.

## Methods

### Participants

We chose the Chinese WeChat users as the source of participants for two reasons. First, WeChat is one of the most popular social media platforms of the world with billions of users. It integrates the dual functions of instant communicating (the main function, like WhatsApp) and personal blogging (the attaching function called the “Moment,” like Facebook). Both the functionality and number of users can ensure that WeChat is the platform that can provide full samples for our study. Second, unlike other social platforms, the main users of WeChat are Chinese, which helps us to exclude the influence of cultural differences when studying PB. Additionally, we also briefly introduced the research objectives, operation process, expected benefits, and ethical considerations, and ensured that all participants were informed and agreed to participate in the current study. Given the concentration of participant sources and the convenience of research (i.e., COVID-19 stops us from handing out questionnaires face to face), we tried to recruit a total of 650 participants through the Credamo platform (www.credamo.com), a reliable Chinese data-collection platform similar to Qualtrics Online Sample (Gong et al., 2020a; Pu et al., 2020), and then paid a certain amount of money in return. To ensure the validity of the 43-item survey, we excluded those abnormal participants who completed the questionnaire within 2 min and more than 10 min. Meanwhile, we applied the favored method, including an item with specific instructions (i.e., asked the participants to select a specific option), to test whether the participants answered thoughtfully (DeSimone et al., 2015). Moreover, we excluded 119 questionnaires with missing essential items and contradictory information (i.e., the reverse and positive items are the same, and all options remain the same). These criteria yielded 497 questionnaires to perform the analysis, with a 76.4% effective rate.

Among the valid participants, in terms of gender, men accounted for 64.59% and women occupied 35.41%. Regarding age, under-20 occupied 10.06%, 20–30 years old 58.55%, 30–40 years old 26.76%, and over 40 years old 4.63%. From the perspective of education, senior high school and below accounted for 36.01, university (junior college and undergraduate) 55.73% with master's and above being 8.26%. Regarding marital status, single (i.e., unmarried, divorced, and widowed) accounted for 52.92% and married accounted for 45.87%. In terms of occupation, students accounted for 26.55%, civil servants 5.04%, public institutions 24.14%, state-owned enterprises 19.91%, private enterprises 21.53%, and foreign-funded enterprises accounted for 2.83%.

### Variable Measurement

To ensure the accuracy of the data analysis, the current study selected a mature scale from the literature of the authoritative journals that have good reliability and validity. Since the participants are Chinese and the original scale comes from English, the scale, according to the procedure of Britain (Brislin, 1980), was translated and back-translated. Table 1 presents the basic quality indicators of variable measurement.

**Table 1:** Means, SDs, reliability, and validity.

**Variable**	**Mean**	**SD**	**Item**	**Cronbach's α**	**KMO**	***p*** **-value**
1. SC	3.816	0.833	17	0.962	0.970	0.000
2. Conversation	4.002	0.841	2	0.783		
3. Advocacy	3.599	1.046	3	0.877		
4. Information	3.827	0.895	4	0.889		
5. Affiliation	3.846	0.94	3	0.860		
6. Utility	3.881	0.918	3	0.864		
7. Identity	3.795	0.962	2	0.811		
8. CS	4.018	0.745	3	0.803	0.712	0.000
9. CNH	3.14	0.837	7	0.866	0.841	0.000
10. PB	4.085	0.685	3	0.726	0.668	0.000

#### Social Currency

The measurement of social currency (SC) scale refers to the scale compiled by Lobschat et al. (2013). The scale includes the following six dimensions: conversation, advocacy, information, affiliation, utility, and identity, with 17 items. It can be seen from Table 1 that all the factors, such as the internal consistency reliability coefficient of each subscale and the overall scale, indicate that the survey has good reliability and validity, which can be employed for further studies.

#### Prosocial Behavior

The PB scale was designed by Jordan et al. (2011). Three items measured prosocial activities (voluntary activities, donation, and blood donation), and three interference items measured entertainment activities (vacation, party, and movie). The participants were asked to rate their willingness to participate in various activities in the coming month (1 represents “very unwilling” and “5” stands for “very willing”). The scale coefficient test of Kaiser-Meyer-Olkin (KMO) was found to be 0.786, and the significant *p-*value was found to be 0, which was < 0.05, explaining 66.49% of the variation. Among them, the three questions on PB belonged to the same factor. The load measured 0.650–0.834, and Cronbach's α coefficient is 0.726. The three questions of entertainment activities belonged to another factor, and the load measured 0.765–0.839, Cronbach's α coefficient is 0.749. It demonstrates that the survey has acceptable reliability and validity, which can be used for further research and analysis. Finally, we dropped the entertainment items and used the items of prosocial activities for summation to obtain the average score of PB.

#### Communication Network Heterogeneity

Based on the recommendation of Kim and Kim (2019), the dimensions of CNH were assessed with demographic and personal factors (Scheufele et al., 2004; Kim et al., 2013). On a 5-point scale from 1 (never) to 5 (very often), the respondents were asked how often they talked /interacted with people of different (a) gender, (b) race, (c) religion, (d) nationality, (e) profession, (f) place of residence, and (g) background. Table 1 demonstrates that the survey has good reliability and validity, which can be employed for further research and analysis.

#### Collective Self-Esteem

As suggested by Kim and Kim (2019), the CS scale was formed based on the deletion of the CS scale compiled by Zhang et al. (2011). The scale consisted of three items, and the subjects scored five points on these items (“1” stands for “very disagree” and “5” denotes “very agree”) with specific items, such as “I am a worthy member of the groups or organizations I come to be an important reflection of who I am.” Table 1 shows that the investigation has good reliability and validity, which can be used for performing further research and analysis.

#### Control Variable

As a gender, age, education level, occupation, and marital status may influence the PB of users, these variables are also included in the questionnaire as control variables.

### Procedures

In the present study, procedural remedial measures and statistical control technology were adopted to ensure the research process. For example, throughout the research, we guaranteed the anonymity of the interviewees and the confidentiality of the questionnaire. Moreover, it needs to point out that the survey results are only used for academic research, and thus, they will be more willing to participate in the research. As a part of the survey, the variables in this study were distributed in different modules and presented in a different order from the research effect, aiming to reduce the possibility of answering questions in accordance with the previous answers.

## Results

### Common Method Biases

Common method variance (CMV) is the overlap of variation among variables caused by the same data sources, measurement tools, and usage environment, while it does not represent the actual relationship between constructions. To minimize the influence of the variation of common methods, in the current study, pre-control measures, such as anonymous filling, establishing antonymous items, and randomly arranging items, were used. Meanwhile, Harman's single factor method is used to identify any common methodological deviations. The results manifest that all items are categorized into four factors whose Eigenvalues are >1. The first factor explains a 32.142% variation of all items, and the threshold value is < 40%. Thus, the current study has better control over the homologous method variation of data. Moreover, in the entire model, the confirmatory factor is tested, and its results show that χ^2^/df = 1.461, confirmatory fit index (CFI) = 0.984, goodness-of-fit index (GFI) = 0.938, root mean square error of approximation (RMSEA) = 0.030, and standardized root mean squared error (SRMR) = 0.043. Once again, the construct validity of all variables in the present study was approved.

### Descriptive Analysis

As presented in Table 2, the results demonstrate that SC is positively associated with PB and its correlation coefficient is 0.408. Moreover, SC is positively correlated with each dimension of PB. The correlation coefficients are 0.731, 0.898, 0.942, 0.921, 0.893, and 0.877. CS and CNH are significantly positively correlated with SC and PB. All these results initially proved our hypothesis.

**Table 2:** Correlation of variables.

**Variable**	**1**	**2**	**3**	**4**	**5**	**6**	**7**	**8**	**9**	**10**
1. SC	1									
2. Conversation	0.731^**^	1								
3. Advocacy	0.898^**^	0.613^**^	1							
4. Information	0.942^**^	0.641^**^	0.827^**^	1						
5. Affiliation	0.921^**^	0.626^**^	0.770^**^	0.864^**^	1					
6. Utility	0.893^**^	0.590^**^	0.720^**^	0.792^**^	0.781^**^	1				
7. Identity	0.877^**^	0.553^**^	0.749^**^	0.764^**^	0.781^**^	0.829^**^	1			
8. CS	0.328^**^	0.338^**^	0.250^**^	0.287^**^	0.319^**^	0.302^**^	0.278^**^	1		
9. CNH	0.425^**^	0.249^**^	0.285^**^	0.262^**^	0.297^**^	0.285^**^	0.274^**^	0.311^**^	1	
10. PB	0.408^**^	0.374^**^	0.324^**^	0.382^**^	0.390^**^	0.372^**^	0.336^**^	0.466^**^	0.365^**^	1

*N = 497*.

** *p < 0.01*.

### Hypothesis Test

This study mainly used SPSS 25.0 to analyze the main effect of SC on PB, the mediating effect of CS, and the moderating effect of CNH through multiple linear regressions. The moderating mediating effect was further tested by PROCESS V3.3.

#### Effect of SC on PB

Hypothesis 1 proposed that SC in social networks has a positive impact on PB. From the results shown in Table 3, the SC has a significant positive effect on PB (M2; β = 0.321, *p* < 0.001), and Hypothesis 1 is verified. For a specific SC, conversation (M3), advocacy (M4), information (M5), affiliation (M6), utility (M7), and identity (M8) have a significant positive effect on the PB of an individual with coefficients of 0.286, 0.203, 0.278, 0.269, 0.264, and 0.227, respectively, all significant at 1% level. Hypothesis 1 is fully supported.

**Table 3:** Regression results of social currency (SC) and prosocial behavior (PB).

**Variable**	**Prosocial behavior**							
	***M*** **1**	***M*** **2**	***M*** **3**	***M*** **4**	***M*** **5**	***M*** **6**	***M*** **7**	***M*** **8**
Age	−0.003	−0.012^*^	−0.022^*^	−0.006^*^	−0.007^*^	−0.004^*^	−0.030	0.008^*^
Gender	0.051	0.059	0.040	0.062	0.059	0.056	0.059	0.060
Education	−0.069	−0.100^*^	−0.093	−0.099^*^	−0.094^*^	−0.092^*^	−0.083^*^	−0.098^*^
Occupation	0.036	0.024	0.019	0.031	0.029	0.022	0.024	0.028
Marital status	−0.261^***^	−0.204^**^	−0.219^***^	−0.227^***^	−0.204^**^	−0.206^**^	−0.217^**^	−0.228^**^
SC		0.321^***^						
Conversation			0.286^***^					
Advocacy				0.203^***^				
Information					0.278^***^			
Affiliation						0.269^***^		
Utility							0.264^***^	
Identity								0.227^***^
*R* ^2^	0.053	0.199	0.170	0.146	0.180	0.183	0.174	0.151
Adjusted *R^2^*	0.043	0.189	0.160	0.135	0.170	0.173	0.164	0.141
*F*	5.485	20.247	16.730	13.930	17.909	18.289	17.249	14.555
*MAX (VIF)*	1.462	1.475	1.470	1.469	1.476	1.475	1.471	1.468

*** *p < 0.001*,

** *p < 0.01*,

* *p < 0.05*.

#### Mediating Effect of CS

Hypothesis 2 proposed that CS plays a mediating role between SC and PB in social networks. This study, referring to the research method conducted by Baron and Kenny (1986), tested the mediating effect of CS. As presented in Table 4, the SC has a significant effect on PB (*M*2, β = 0.321, *p* < 0.001). SC has a significant influence on CS (*M*10, β = 0.289, *p* < 0.001). SC and CS have significant effects on PB (*M*12, β = 0.227, 0.327, respectively, *p* < 0.001). Thus, it can be proved that the mediating effect of CS is significant. Hypothesis 2 is partly supported.

**Table 4:** Results of mediating effects of collective self-esteem.

**Variable**	**Collective self-esteem**		**Prosocial behavior**			
	***M*** **9**	***M*** **10**	***M*** **1**	***M*** **2**	***M*** **11**	***M*** **12**
Age	0.010	0.002	−0.003	−0.012^*^	−0.007	−0.012
Gender	0.069	0.076	0.051	0.059	0.023	0.034
Education	−0.024^*^	−0.052^*^	−0.069	−0.100^*^	−0.060	−0.083
Occupation	0.005	−0.006	0.036	0.024	0.034	0.026
Marital status	−0.188^*^	−0.136	−0.261^***^	−0.204^**^	−0.185^**^	−0.160^**^
SC		0.289^***^		0.321^***^		0.227^***^
CS					0.407^***^	0.327^***^
*R* ^2^	0.025	0.124	0.053	0.199	0.245	0.310
Adjusted *R^2^*	0.015	0.114	0.043	0.189	0.235	0.300
*F*	2.479	11.600	5.485	20.247	26.448	31.346
*MAX (VIF)*	1.462	1.475	1.462	1.475	1.479	1.485

*** *p < 0.001*,

** *p < 0.01*,

* *p < 0.05*.

#### Moderating Effect of CNH

Hypothesis 3 proposed that CNH plays a positive moderating role between SC and CS in social networks. To reduce the multicollinearity in the stepwise multiple linear regression progress, all variables were centralized before the analysis. The results of the analysis are displayed in Table 5. Regarding the significant positive correlation between SC and CS, the CNH plays a significant positive role (*M*14, β = 0.091, *p* < 0.001). Hypothesis 3 is supported.

**Table 5:** Results of moderating effect of communication network heterogeneity (CNH).

**Variable**	**Collective self-esteem**			
	***M*** **9**	***M*** **10**	***M*** **13**	***M*** **14**
Age	0.010	0.002	−0.083	−0.065
Gender	0.069	0.076	0.032	0.049
Education	−0.024^*^	−0.052^*^	−0.020	−0.049
Occupation	0.005	−0.006	−0.017	−0.019
Marital status	−0.188^*^	−0.136	−0.122	−0.103
SC		0.289^***^		0.289^***^
CNH			0.376^***^	0.218^***^
SC * CNH				0.091^***^
*R* ^2^	0.025	0.124	0.191	0.254
Adjusted *R^2^*	0.015	0.114	0.181	0.241
*F*	2.479	11.600	19.324	20.717
*MAX (VIF)*	1.462	1.475	1.474	1.409

*** *p < 0.001*,

* *p < 0.05*.

**Figure 2:**
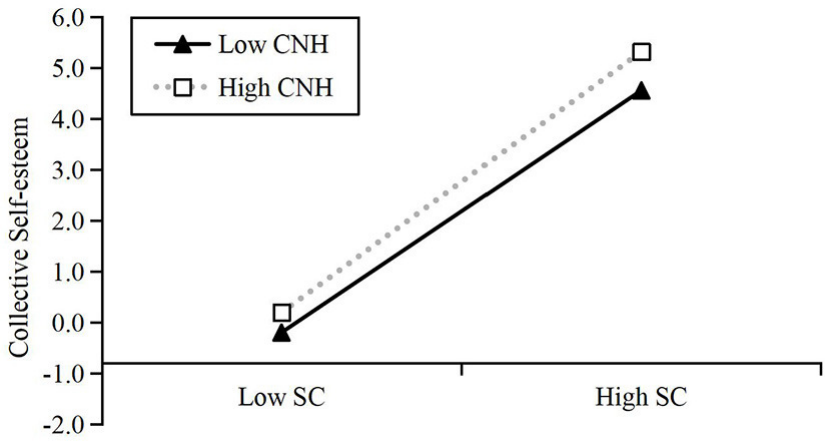
The moderating effect plot of communication network heterogeneity (CNH).

Besides, to further understand the relationship between SC and CS, we drew a diagram based on the method and procedure proposed by Aiken et al. (1991). According to the standard of high/low average and a moderating effect plot drawn, all these factors were divided into two groups including high (M+1SD) and low (M-1SD). As can be seen in the plot of this moderating effect in Figure 2, the higher the level of CNH is, the stronger is the positive effect of SC on CS. Therefore, the CNH plays a positive moderating role between SC and CS. This result further supports Hypothesis 3 of the present study.

#### The Moderated Mediating Effect

Hypothesis 4 proposed that the higher the CNH is, the stronger is the mediating role of CS between SC and individual PB, and vice versa. The conditional indirect effect at different values of the moderating variable was obtained directly in this study by the PROCESS operation. The PROCESS automatically operates this different value as a decrease of one SD and an increase of one SD from the mean value of the moderating variable, constituting the low and high values, respectively. As can be seen from the results displayed in the left part of Table 6, when the CNH of individuals is low, the indirect effect of SC affecting PB through CS is found to be 0.036 [confidence interval (−0.003, 0.083), including 0], so the indirect effect is not significant. When the CNH of individuals was high, the indirect effect of SC influencing PB through CS was 0.107 [confidence interval (0.060, 0.172, excluding 0)], with a significant indirect effect.

**Table 6:** Moderated mediating effect.

**Moderator variable**	**SC (X) → Collective self-esteem (M) → Prosocial behavior (Y)**							
	**Indirect effect**				**Moderated mediating effect**			
	**Effect**	**SE**	**LLCI**	**ULCI**	**Index**	**SE**	**LLCI**	**ULCI**
Low CNH	0.036	0.022	−0.003	0.083	0.043	0.020	0.005	0.085
High CNH	0.107	0.029	0.060	0.172				

*LLCI, lower limit confidence interval; ULCI, upper limit confidence interval*.

Thus, in this case, the analysis of conditional indirect effects alone is not sufficient to determine whether there is a moderating mediating effect. Therefore, the right-hand part of Table 6 highlights the INDEX indicator obtained from the PROCESS operation; it can be seen that the indirect relationship between SC and PB through CS has a moderating effect of 0.043 [confidence interval (0.005, 0.085), excluding 0], thus indicating that moderated mediating effects are significant. This result fully supports Hypothesis 4.

**Figure 3:**
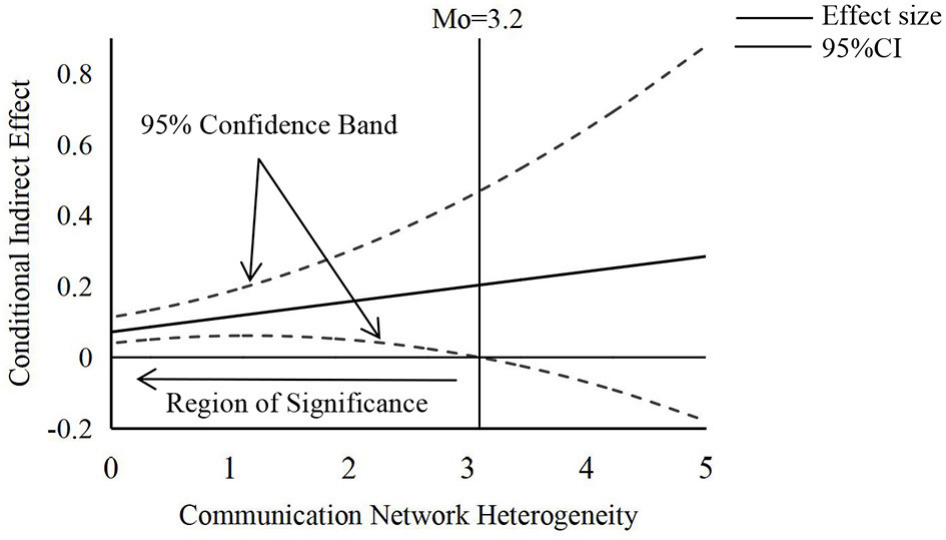
The moderated mediating effect plot of CNH.

Indeed, the moderating mediating effect of the current study is a linear function of the moderating variable. However, prior tests (e.g., group analysis and analysis of variance) can capture indirect effects only for two distinct values of the moderating variables and so do not completely reflect the indirect effects impacted by the moderating variables (Yu et al., 2021). To address this shortcoming, this study analyzed the conditional indirect effects under continuous values of the moderating variable by calculating the specific values of the 95% confidence bands and the region of significance using the Johnson-Neyman method provided by PROCESS V3.3 (Hayes, 2017), as Preacher et al. (2007) did. In Figure 3, the straight line denotes the moderating impact on the dependent variable, which is a linear function of the moderating variable, while the dashed line denotes the equivalent confidence band. As can be seen in Figure 3, the indirect effect of SC on individual PB through CS is significant when the value of CNH is < 3.20 (5 is a perfect score). The results of the study further support Hypothesis 4.

## Conclusion and Implications

### Discussion

To reveal the influence mechanism of PBs on social networks, the present study constructed a model of the effect of the SC of users on PBs in social networks mediated by CS and moderated by the CNH. Additionally, it conducted empirical research with the following findings.

To begin with, the SC of users in social networks has a positive effect on PB, so Hypothesis 1 is supported. To the best of our knowledge, this study is one of the earliest studies to examine the relationship between SC and PB. Consistent with previous research findings, communal elements contribute significantly to shaping rich experiences (Srivastava and Kaul, 2014; Piyathasanan et al., 2015). Notably, our research demonstrates that SC is a critical predictor of PB and that the more SC the users possess, the more likely they are to act prosocially on their own. By sharing their own life experiences and fostering PB with the outside world, the users can ensure the “input” and “output” of information in social networks, as well as the “continuous appreciation” of SC. To be completely candid, this interaction contributes to a better understanding of information. It encourages sharing and replicating its essential components, empathic connections with the stories of other people and organizations, and developing a sense of belonging and emotional connection. When positive elements accumulate, they exhibit behaviors that are beneficial to individuals, teams, and society. For instance, preliminary research indicates that SC plays a significant role in the brand experience (Trudeau and Shobeiri, 2016). Businesses provide feedback and comments that expose individuals to a variety of experiences and even motivate them to adapt to their lifestyles. As a result, they may more effectively adhere to the social model of the group.

There is one more point which we should touch on, that is, CS plays a mediating role between the SC of users and PB, so Hypothesis 2 is supported. Previously, the underlying mechanism between PB and its antecedents has been usefully explored while it is more concerned about the mechanisms of the action of family factors (e.g., the company of parents and parenting), social factors (e.g., social support and social identity), and individual factors (motive power, nostalgia, and pride). While some scholars have discussed the mediating role of CS in the partial relationship between user SC and PB, the current study builds on and expands on that discussion. Specifically, this study has enriched the findings of Fu et al. (2017), who argued that a variety of factors, including emotional factors, self-esteem, and empathy, are associated directly or indirectly with PB. Additionally, this study asserts that users can influence PB by affecting their CS in SC, and thus, the perceptions of the users on CS (individual cognitive factors) may be viewed as a mediating variable affecting these linkages. Additionally, the obtained conclusion serves as a guide for social managers seeking to improve the CS of the users.

In the third place, the CNH plays an important moderating role in the process of the SC of the users on CS, so Hypothesis 3 is supported. Some previous studies revealed that the level of group identity and CS may vary depending on individual characteristics (Crocker et al., 1994). To be consistent with them, the results of the present study give information about when the CNH in social networks is higher, and the linkage of SC and CS becomes more obvious. In other words, if individuals within their social networks continue to share, listen, and interact, they may have a high level of heterogeneity. Therefore, they would feel a sense of community belonging, pay more attention to words and deeds in real life, maintain the social image of the community, and provide favorable conditions for the formation of CS. Most notably, our line of research is inconsistent with Kim and Kim (2019), who consider communication network heterogeneity as a mediating variable between social media use and collective self-esteem, whereas we treat communication network heterogeneity as a boundary condition for the effect of social currency on collective self-esteem.

Finally, CNH moderates the mediating effect of CS between SC and PB, so Hypothesis 4 is supported. For those with a highly heterogeneous communication network, when the SC in the social network enables complete communication with the outside world, deepens collective social self-emotion, and establishes CS, it will be applied to social network resources to complete the collective social task and realize the PB of the group or the organization.

### Conclusion

To conclude based on the SET, this study empirically validates how SC promotes the PB of individuals through CS in social networks, and draws three-fold conclusions. (1) Exploring the direct effect of SC on individual PB in social networks. SC in social networks may influence individuals to perform PB, that is, the more the SC users perceive, the more likely are they to behave better in the group, organization, and society. (2) Illustrating the internal effect mechanism of SC on individual PBs in social networks. The SC will promote the possibility of the CS perception of individuals in their social networks, thus enhancing the probability of the PBs of the users. (3) Clarifying the effect mechanism of boundary conditions. The CNH may positively influence the relations of SC and CS, and strengthen the path effect of SC on individual PB, namely, the higher CNH is, the greater is the influence of SC on CS among social network users and the stronger is the influence path of SC on individual PB.

Although the present study provides theoretical and practical implications, there are still some limitations requiring to be addressed in future research. To begin with, this study only examines the relationship between SC and PB using a single cognitive and affective mechanistic factor (CS). However, SC, as a situational stimulus, may also stimulate other cognitive factors (such as social identity, social distance, social presence, and trust) and emotional factors (such as empathy and pleasure) that may influence PB. In addition, only one individual factor as a moderator variable is discussed in the present study. To further elucidate the theoretical model, future research can examine additional cognitive-emotional factors that mediate the relationship between SC and PB, as well as other factors that may be moderated along the mediation path (such as information content type and user involvement). Second, we adopted the scale of foreign literature, which develop the western cultural background and thinking mode. Although we have adopted a strict yardstick, referred to two-way translation, and modified it again before testing and consulting experts, the differences in the Eastern culture are not fully considered, maybe leading to missing the influence factors of the cultural characteristics in social networks. In future studies, the scale can be developed based on the Chinese context to ensure its validity. Third, this study employed a cross-sectional survey method, only finding the concurrent relationship among variables. Meanwhile, due to data collection via a self-report, the respondents might hide their real information, and the study itself will be influenced by numerous unexpected factors, such as the interference of a problem-solving environment and mood swings. Future studies can measure variables more accurately through various measurement methods (e.g., eye movement experiments and event-related potential methods) and attempt to employ longitudinal tracking or situational experiment to analyze the deeper causal relationship among variables.

## Data Availability Statement

The raw data supporting the conclusions of this article will be made available by the authors, without undue reservation.

## Ethics Statement

The studies involving human participants were reviewed and approved by Southwestern University of Finance and Economics Ethics Committee. The patients/participants provided their written informed consent to participate in this study.

## Author Contributions

XG and ZY designed the research framework and wrote the manuscript. BL and YY analyzed the data. SY and YY edited the manuscript. All authors contributed to the article and approved the submitted version.

## Conflict of Interest

The authors declare that the research was conducted in the absence of any commercial or financial relationships that could be construed as a potential conflict of interest.

## Publisher's Note

All claims expressed in this article are solely those of the authors and do not necessarily represent those of their affiliated organizations, or those of the publisher, the editors and the reviewers. Any product that may be evaluated in this article, or claim that may be made by its manufacturer, is not guaranteed or endorsed by the publisher.
